# Activation of the MEK-ERK Pathway Is Necessary but Not Sufficient for Breaking Central B Cell Tolerance

**DOI:** 10.3389/fimmu.2018.00707

**Published:** 2018-04-09

**Authors:** Sarah A. Greaves, Jacob N. Peterson, Raul M. Torres, Roberta Pelanda

**Affiliations:** ^1^Department of Immunology and Microbiology, University of Colorado School of Medicine, Aurora, CO, United States; ^2^Department of Biomedical Research, National Jewish Health, Denver, CO, United States

**Keywords:** B cells, B cell tolerance, BCR signaling, MAP kinase, ERK, B cell development, autoreactive B cells, mouse models

## Abstract

Newly generated bone marrow B cells are positively selected into the peripheral lymphoid tissue only when they express a B cell receptor (BCR) that is nonautoreactive or one that binds self-antigen with only minimal avidity. This positive selection process, moreover, is critically contingent on the ligand-independent tonic signals transduced by the BCR. We have previously shown that when autoreactive B cells express an active form of the rat sarcoma (RAS) oncogene, they upregulate the receptor for the B cell activating factor (BAFFR) and undergo differentiation *in vitro* and positive selection into the spleen *in vivo*, overcoming central tolerance. Based on the *in vitro* use of pharmacologic inhibitors, we further showed that this cell differentiation process is critically dependent on the activation of the mitogen-activated protein kinase kinase pathway MEK (MAPKK)-extracellular signal-regulated kinase (ERK), which is downstream of RAS. Here, we next investigated if activation of ERK is not only necessary but also sufficient to break central B cell tolerance and induce differentiation of autoreactive B cells *in vitro* and *in vivo*. Our results demonstrate that activation of ERK is critical for upregulating BAFFR and overcoming suboptimal levels of tonic BCR signals or low amounts of antigen-induced BCR signals during *in vitro* B cell differentiation. However, direct activation of ERK does not lead high avidity autoreactive B cells to increase BAFFR levels and undergo positive selection and differentiation *in vivo*. B cell-specific MEK-ERK activation in mice is also unable to lead to autoantibody secretion, and this in spite of a general increase of serum immunoglobulin levels. These findings indicate that additional pathways downstream of RAS are required for high avidity autoreactive B cells to break central and/or peripheral tolerance.

## Introduction

B cells developing in the bone marrow rearrange their immunoglobulin heavy (IgH) and immunoglobulin light (IgL) chain genes in order to form B cell receptors (BCRs) specific for a wide array of pathogens. Due to its random nature, this process also produces B cells that are reactive with self-antigens (i.e., autoreactive B cells). In fact, a large majority (~55–75%) of immature B cells that are newly generated in the bone marrow of mice and people are autoreactive (1, 2). In a healthy immunological system, these autoreactive immature B cells are eliminated or controlled by mechanisms of negative selection known as B cell tolerance (1, 3–5). About half of these cells, those with BCRs with low avidity for self-antigen, enter the circulation and undergo further differentiation but are rendered anergic in the periphery and die shortly thereafter [reviewed in Ref. (6, 7)]. The other half, those with medium/high avidity BCRs for self-antigens, continue rearranging their IgL chain genes to form a new BCR, a central tolerance mechanism known as receptor editing (1, 3–5). Once these editing B cells produce a nonautoreactive (NA) BCR, a “tonic” ligand-independent signal is generated (8, 9). Our lab and others have shown that this tonic signal is crucial for the positive selection of B cells into the spleen, their differentiation into transitional and mature cell stages, and their long-term survival in the periphery (5, 10–12).

B cell repertoire studies have shown that in patients affected by some autoimmune diseases (including systemic lupus erythematosus), an increased number of autoreactive B cells escape from the bone marrow into the periphery to play a role in disease (13–15). In addition, many B cells participating in disease flares in lupus patients carry germline encoded Ig variable region sequences, suggesting they are direct descendants of B cells that escaped central tolerance (16). This has also been demonstrated in mice in which B cells expressing germline encoded Ig genes contribute to anti-nuclear antibody production (17). Determining the molecular mechanisms of how autoreactive and NA immature B cells undergo positive selection into the periphery is, therefore, of great importance in order to establish who is at risk for autoimmune diseases. This knowledge may also lead to the development of new therapies that restrict the bone marrow output of autoreactive B cells, thus decreasing the risk for the onset of these diseases. However, the molecular mechanisms that lead autoreactive B cells to break central tolerance are still largely unknown.

Rat sarcoma (RAS) is a small GTPase involved in many fundamental cellular processes, including cell differentiation and survival (18). RAS is thought to be the main activator of the extracellular signal-regulated kinase (ERK) pathway, *via* the intermediate MAP kinases RAF and MEK, all of which are also essential cell signaling components (19). In previous studies we have shown that basal activation of both RAS and ERK is higher in NA than autoreactive immature B cells of mouse models of central tolerance (20, 21). In addition, NA immature B cells bearing hypomorphic BCR levels with reduced tonic signaling (BCR-low cells) exhibit low levels of active RAS and ERK that are similar to those of autoreactive cells (20, 21). We have further shown that inhibition of the MAPK MEK-ERK pathway in NA immature B cell cultures prohibits cell differentiation into the transitional stage (20, 21). Taken together, these data have revealed a positive correlation between surface BCR levels and intracellular activity of the RAS-ERK pathway in immature B cells and have also indicated that basal activation of the ERK pathway is necessary for propagation of tonic BCR signaling and the differentiation of immature B cells into transitional B cells.

Heightened levels of phospho-ERK (pERK) have been observed in B cells from both lupus patients and some lupus mouse models (22–24) suggesting that this pathway contributes to the generation and/or the survival and activation of autoreactive B cells. In support of this idea, we have shown that expression of a constitutively active form of NRAS (caNRAS) in NA BCR-low and in autoreactive immature B cells increases their basal pERK levels, inhibits receptor editing *in vitro* and *in vivo*, promotes *in vitro* cell differentiation, and, in some instances, induces the *in vivo* production of IgG autoantibodies (20, 21). Because activation of the MEK-ERK pathway is downstream of RAS, this has led us to hypothesize that activation of the ERK pathway is not only necessary but may also be sufficient to overcome defects in BCR tonic signaling or the presence of self-antigen-induced BCR signaling and, consequently, to promote the differentiation of NA BCR-low and autoreactive B cells. To our knowledge, whether activation of the ERK pathway overcomes B cell tolerance has never been tested.

To test this hypothesis, in this study, we used a gene cassette encoding a constitutively active form of MEK (caMEK) either as a retroviral-driven transgene in bone marrow cultures or as a Cre-regulated Rosa-26 targeted locus *in vivo*. This latter approach has been successfully employed to investigate the signaling pathways that ensure the BCR-dependent survival of mature B cells (25). Using these approaches we found that in bone marrow B cell cultures, ERK activation can overcome suboptimal levels of tonic BCR signals or low amounts of antigen-induced BCR signals to promote the differentiation of BCR-low or autoreactive immature B cells into transitional B cells. However, direct activation of the MEK-ERK pathway is unable to break B cell tolerance *in vivo*, neither preventing receptor editing nor allowing cell differentiation of high avidity autoreactive B cells. These findings indicate that activation of ERK is not sufficient to break central B cell tolerance and that additional pathways downstream of RAS are required for this outcome.

## Materials and Methods

### Mice

Ig knock-in mice 3-83Igi,H-2^d^ (NA), 3-83Igi,H-2^d^-low (NA with low BCR), and 3-83Igi,H-2^b^ (autoreactive) have been previously described (20, 26–29) and were all on a BALB/c genetic background. BALB/c mb1-Cre mice described in Ref. (30) were kindly donated by Michael Reth (Max Planck Institute of Immunobiology and Epigenetics) and were bred to 3-83Igi,H-2^b^ mice to generate 3-83Igi,H-2^b^-mb1-Cre mice. CB17 mice, bred in house, were used as wild-type controls. BALB/c mice (Stock No. 000651) were purchased from The Jackson Laboratory. The Rosa26-Lox-stop-Lox (LSL)-caMEK-GFP mice previously described in Ref. (25) were purchased from The Jackson Laboratory (Stock No. 012352) and were backcrossed to CB17,H-2^b^ mice (4 generations) and then bred to 3-83Igi,H-2^b^ mice (eight generations) to generate 3-83Igi,H-2^b^-R26-LSL-caMEK-GFP mb1-cre mice. Mice were analyzed at 7–9 weeks of age. Mice were bred and maintained in a specific pathogen-free facility at the University of Colorado Anschutz Medical Campus Vivarium (Aurora, CO, USA). Both male and female mice were used for experiments, and all animal procedures were approved by the UCD Institutional Animal Care and Use Committee.

### Retroviral Constructs and Production of Retroviral Particles

The following retroviral vectors encoding replication-deficient retroviruses were used: pMSCV-IRES-GFP (MIG) and pMSCV-GFP-IRES-hN-RasG12D (NRAS) were previously described (20). We obtained a shuttle vector containing constitutively active form of MEK [caMEK, MEK1-S218E/S222D (31)] from Addgene (plasmid #40809). The caMEK gene cassette was amplified from this plasmid using the primers caMEK-NotI (5′-AAAGCGGCCGCGTTACCCGGGTCCAAAA-3′) and caMEK-SalI (5′-AATGTCGACTTAGACGCCAGCAGCATG-3′) and AccuTaq polymerase (Sigma). The PCR product was cloned between NotI and SalI in the retroviral pMSCV-IRES-GFP vector (20) to generate the pMSCV-caMEK-IRES-GFP plasmid. These vectors are based on the Murine Stem Cell Virus (MSCV) retroviral expression system and contain an internal ribosome entry site (IRES) for bicistronic gene expression. We obtained the ERK2 shRNA and luciferase shRNA sequences in shuttle vectors from Open Biosystems. The shRNA sequences were isolated from these vectors as XhoI-EcoRI fragments that were then cloned into the MSCV-LTRmiR30-PIG (LMP) vector (Open Biosystems), kindly donated by John Cambier (University of Colorado), to create the MSCV-LMP-ERK2-shRNA and MSCV-LMP-luc-shRNA plasmids. Retroviral particles were produced as described previously (20).

### *In Vitro* Immature B-Cell Differentiation and Transduction

Bone marrow immature B cells were generated and differentiated *in vitro* as previously described (20, 21) based on a B cell culture system originally described in Ref. (32). Briefly, bone marrow cells were cultured in complete Iscove’s Modified Dulbecco’s Medium in the presence of IL-7 (made in house) for 4 days at which time IL-7 was removed by washing twice with PBS. Then, cells were plated at 6–8 × 10^6^ cells/mL with 10 ng/mL recombinant mouse BAFF (R&D Systems) for an additional 2–3 days to achieve cell differentiation (e.g., CD21 and IgD expression). Where indicated, cells were treated with either DMSO, 30 µM of ERK1/2 inhibitor (FR180204; EMD Chemicals), or indicated concentrations of anti-3-83Ig idiotypic antibody S23 (33), during culture with BAFF. S23 was added to the culture each day in order to maintain BCR engagement. Retroviral transduction of immature B cells was performed as previously described (20).

### ELISAs

The 3-83IgM and total IgM serum titers were measured by ELISA as previously described (29). The 3-83IgG2a serum titer was measured by ELISA as previously described (29) and with the following modifications. Briefly, 96-well Nunc- Immuno MaxiSorp plates (Thermo Fisher Scientific) were coated with 10 µg/mL of rat anti-mouse IgG2a (RMG2a-62) (purchased from BD Pharmingen). The 3-83IgG was detected using biotinylated anti-3-83Ig antibody (54.1) (34), followed by alkaline phosphatase (AP)-conjugated streptavidin (SouthernBiotech), and developed by the addition of AP substrate (*p*-nitro-phenyl phosphate; Sigma). For total IgG ELISA, 96-well Nunc-Immuno MaxiSorp plates were coated with 10 µg/mL of goat anti-mouse IgG (H+L) antibody, human ads-unlabeled (SouthernBiotech). Plates were detected with goat anti-mouse IgG, human ads-AP (SouthernBiotech). The standard used to measure total IgG concentration was a mixture of the following mouse unlabeled antibodies starting at 1 μg/mL: IgG1 (15H6), IgG2a (HOPC-1), IgG2b (A-1), IgG3 (B10), all purchased from Southern Biotech. For the total IgA ELISA, plates were coated with 10 µg/mL unlabeled goat anti-mouse IgA antibody (SouthernBiotech) and detected with goat anti-mouse IgA-AP antibody (SouthernBiotech). For the total IgE ELISA, plates were coated with 10 µg/mL rat anti-mouse IgE antibody (23G3, SouthernBiotech), and detected with rat anti-mouse IgE-AP antibody (23G3, SouthernBiotech). The standards used to measure sera concentrations are as follows: mouse IgA-unlabeled (S107, SouthernBiotech, starting at 1 µg/mL), mouse IgE-unlabeled (15.3, SouthernBiotech, starting at 0.2 µg/mL). All ELISA plates were developed by the addition of AP substrate (Sigma) solubilized in 0.1 M diethanolamine and 0.02% NaN_3_. Plates were read as previously described (35).

### Generation of Retrovirus-Transduced Mixed Bone Marrow Chimeras

Bone marrow chimeras with transduced hematopoietic stem cells (i.e., retrogenic) were generated as previously described (36) with the following modifications. Briefly, donor 3-83Igi,H-2^d^-low bone marrow cells were transduced with two cycles of spin infection as previously described (36). Recipient BALB/c mice were lethally irradiated with two doses of 450 rad, 3 h apart. They then received a total of 5 × 10^5^ donor cells mixed at the indicated ratios in PBS *via* tail vein injection. Mice were analyzed 8–9 weeks later.

### Quantitative Real-Time PCR

*Ex vivo* bone marrow B cells (either B220^+^ or B220^+^GFP^+^) were isolated using a FACSAria (BD Biosciences) cell sorter with a purity of >97%. Total RNA was purified using TRIzol (Invitrogen) and cDNA was synthesized using the SuperScript III First-Strand Synthesis system (Invitrogen). Murine *Rag1* (Mm01270936_m1) and *Rag2* (Mm00501300_m1) cDNAs were amplified using Applied Biosystems TaqMan primer and probe sets purchased from Thermo Fisher Scientific. Differences in specific mRNA levels were determined as previously described (21), and each sample was normalized to murine 18 s (Mm03928990_g1, AB TaqMan). All samples were run in triplicate using the LightCycler 480 instrument (Roche).

### Flow Cytometry

Bone marrow and spleen single-cell suspensions were stained with fluorochrome or biotin-conjugated antibodies against mouse B220 (RA3-6B2), IgD (11-26c-2a), IgM^a^ (MA-69), IgM^b^ (AF6-78), pan-IgM (11/41), CD21 (7E9), CD23 (B3B4), CD24 (M1/69), Igλ (RML-42), CD19 (1D3), CD138 (281-2), CD86 (B7-2), CD69 (H1.2F3), CD1d (1B1), and CD44 (1M7) purchased from eBioscience, BD Pharmingen, or Biolegend. Anti-3-83Ig (H + κ) antibodies [54.1 (34)] were produced in house. Biotin-labeled antibodies were visualized with flourochrome-conjugated streptavidin (BD Biosciences). The fluorescent chemical compound 7-aminoactinomycin D (7AAD; eBioscience) was used to discriminate unfixed dead cells. Phospho-ERK and total ERK staining were performed on cells treated with the phosphatase inhibitor pervanadate for 5 min as previously described (20, 21). Zombie UV Fixable Viability kit from BioLegend was used to discriminate dead cells in fixed and permeabilized samples. Data acquisition was done on the CyAn cytometer (Beckman Coulter) or the BD LSRFortessa cytometer (BD Biosciences) and analyzed with FlowJo software (Tree Star). Analyses were performed on live B cells based on the incorporation of either 7AAD or Zombie UV and the pan B-cell marker B220 expression. Cell doublets were excluded based on the side scatter and pulse width for data analyzed on the Cyan cytometer or the forward scatter area and forward scatter height for data analyzed on the BD LSRFortessa.

### Statistical Data Analysis

Data were analyzed using GraphPad Prism software. Statistical significance was assessed using an unpaired, one-tail, Student’s *t*-test. *P*-values of ≤0.05 were considered significant. Data are represented as mean ± SEM.

## Results

### ERK Contributes to Establishing Appropriate Expression of the BCR and BAFFR on Immature B Cells

Our previous studies have indicated that basal activation of the ERK pathway is necessary for the differentiation of immature B cells into transitional B cells. These studies, however, were performed with pharmacologic inhibitors of the ERK pathway that, due to potential off target effects, may lead to erroneous conclusions [e.g., (37)]. In order to confirm the requirement for ERK in the differentiation of transitional B cells, we expressed an shRNA specific for ERK2 [the ERK isoform more highly expressed in immature B cells (21)] in NA bone marrow B cells from the Ig knock-in mouse model 3-83Igi,H-2^d^ (26, 28), and then measured their differentiation into CD21^+^ transitional B cells (Figure S1A in Supplementary Material). Expression of ERK2-shRNA led to lower levels of total ERK and of pERK in immature B cells, levels that were particularly reduced in a fraction of transduced cells (Figures S1B,C in Supplementary Material). In agreement with previous observations obtained with the use of MEK and ERK inhibitors (20, 21), knock-down of ERK2 was accompanied by a decreased frequency of CD21^+^ transitional B cells and, again, with a noticeable fraction expressing lower levels of CD21 (Figure S1D in Supplementary Material).

**Figure 1:**
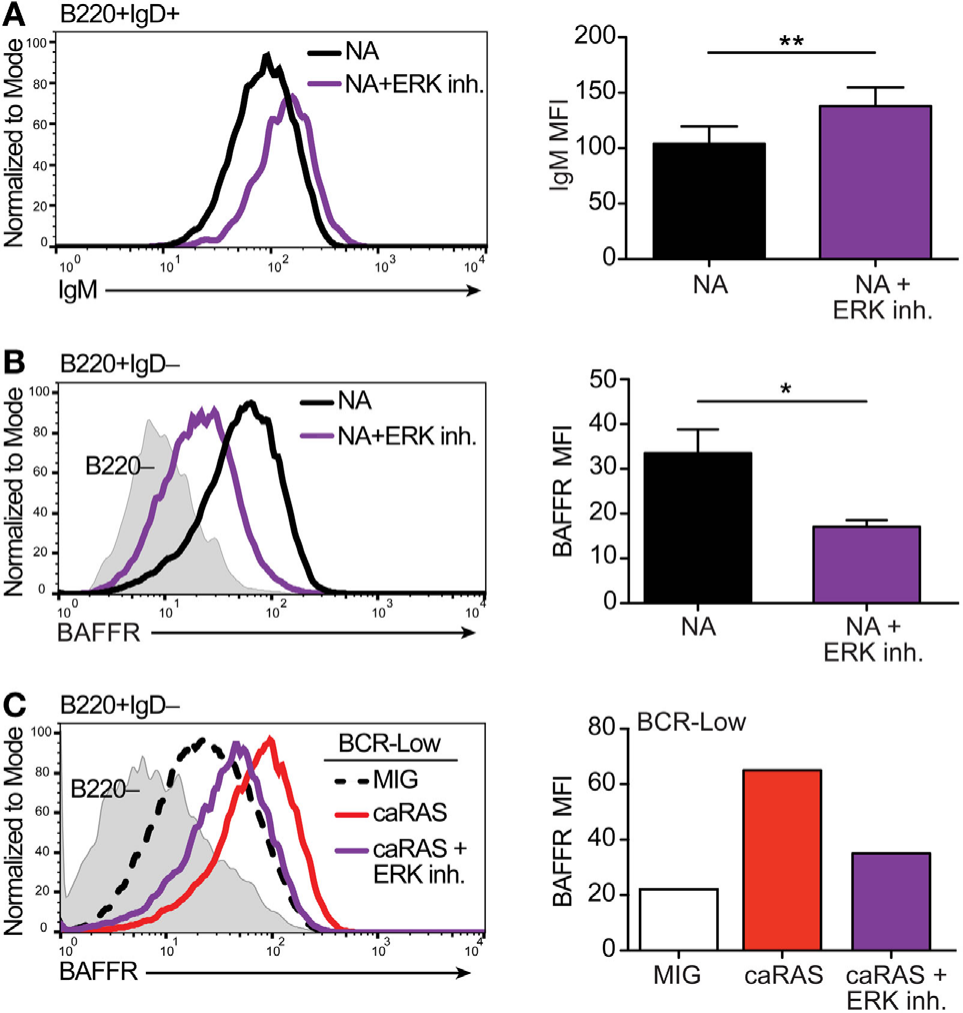
Extracellular signal-regulated kinase (ERK) contributes to establishing appropriate expression of the BCR and BAFFR on immature B cells. **(A)** Representative histograms and quantification of IgM levels on nonautoreactive (NA) (3-83Igi,H-2^d^) bone marrow cells that were cultured in IL-7 for 4 days and then incubated with either BAFF + DMSO (black) or BAFF + 30 µM ERK1/2 inhibitor (FR180204, purple) for 3 days. Shown are B220^+^IgD^+^ transitional B cells. *N* = 4 total, from four independent experiments. **(B)** Representative histograms and quantification of BAFFR levels on NA bone marrow cells that were cultured in IL-7 for 4 days along with either DMSO (black) or 30 µM ERK1/2 inhibitor (purple). Shown are B220^+^IgD^–^ immature B cells. *N* = 3 total, from three independent experiments. **(C)** Representative histograms and quantification of BAFFR levels on BCR-low bone marrow cells that were cultured in IL-7 for 4 days and transduced with either MIG control or caRAS and then incubated with either DMSO (MIG, black dashed line or white bar and caRAS, red) or 30 µM ERK1/2 inhibitor (caRAS, purple). Data are representative of two independent experiments. *P ≤ 0.05, **P ≤ 0.01.

Results from our previous studies (20, 21) have led us to suggest that basal ERK activation is under the control of tonic BCR signaling in immature B cells and that a level of pERK above a set threshold is required for the differentiation of immature B cells into transitional B cells. In order to test this idea, we treated NA bone marrow B cell cultures with an ERK inhibitor, and measured surface IgM on cells that differentiated into IgD^+^ transitional B cells. Pharmacologic inhibition of ERK led to the differentiation of fewer IgD^+^ transitional B cells (data not shown), and the cells that were able to differentiate displayed higher surface IgM levels when compared to control treated cells (Figure 1A). These data support the idea that a threshold of pERK under the control of surface IgM is required to promote ongoing immature B cell differentiation.

BAFFR expression and signaling contributes to the differentiation of immature into transitional B cells and of transitional into mature B cells (36, 38–40). Moreover, BAFFR is expressed at higher RNA and protein levels by NA immature B cells relative to BCR-low and autoreactive B cells (36). Therefore, we next asked if the activity of ERK is necessary for BAFFR expression by immature B cells. Upon treating cultures of NA bone marrow immature B cells with an ERK inhibitor we found significantly reduced expression of BAFFR relative to control (Figure 1B), suggesting that ERK activity contributes to the expression of BAFFR and the response of immature B cells to BAFF. Our previous studies have shown that the expression of caNRAS restores BAFFR levels on the surface of BCR-low immature B cells in culture (20, 36). Given that RAS is a potent activator of the ERK pathway (41), we questioned whether the upregulation of BAFFR mediated by caNRAS (36) also requires ERK activity. To test this, we transduced BCR-low immature B cell cultures with caNRAS and then treated the cells with an ERK inhibitor. Inhibition of ERK almost completely prevented the induction of BAFFR expression mediated by caNRAS (Figure 1C).

Taken together, these data reinforce the conclusion that under the control of surface IgM and tonic BCR signaling, the ERK pathway plays a crucial role in the differentiation of immature B cells into transitional B cells and, thus, for the establishment of central B cell tolerance. They also indicate a critical role of ERK downstream of RAS in the upregulation of BAFFR, an event that contributes to the generation of transitional B cells in mice. We next asked whether direct activation of the ERK pathway is also sufficient to overcome defective tonic signals and to break central B cell tolerance.

### ERK Activation Overcomes Suboptimal Levels of Tonic BCR Signals or Low Amounts of Antigen-Induced BCR Signals During the *In Vitro* Differentiation of Immature B Cells Into Transitional B Cells

To determine if ERK activation alone could facilitate BAFFR expression to levels similarly driven by caNRAS and could also promote the differentiation of BCR-low B cells, we conducted *in vitro* experiments with a constitutively active form of MEK (caMEK), the kinase that directly activates ERK. A gene encoding caMEK [MEK1-S218E/S222D (31)] was cloned into a retroviral vector that includes an IRES-GFP cassette, and caMEK-IRES-GFP retroviral particles were transduced into BCR-low immature B cells in an IL-7 culture.

**Figure 2:**
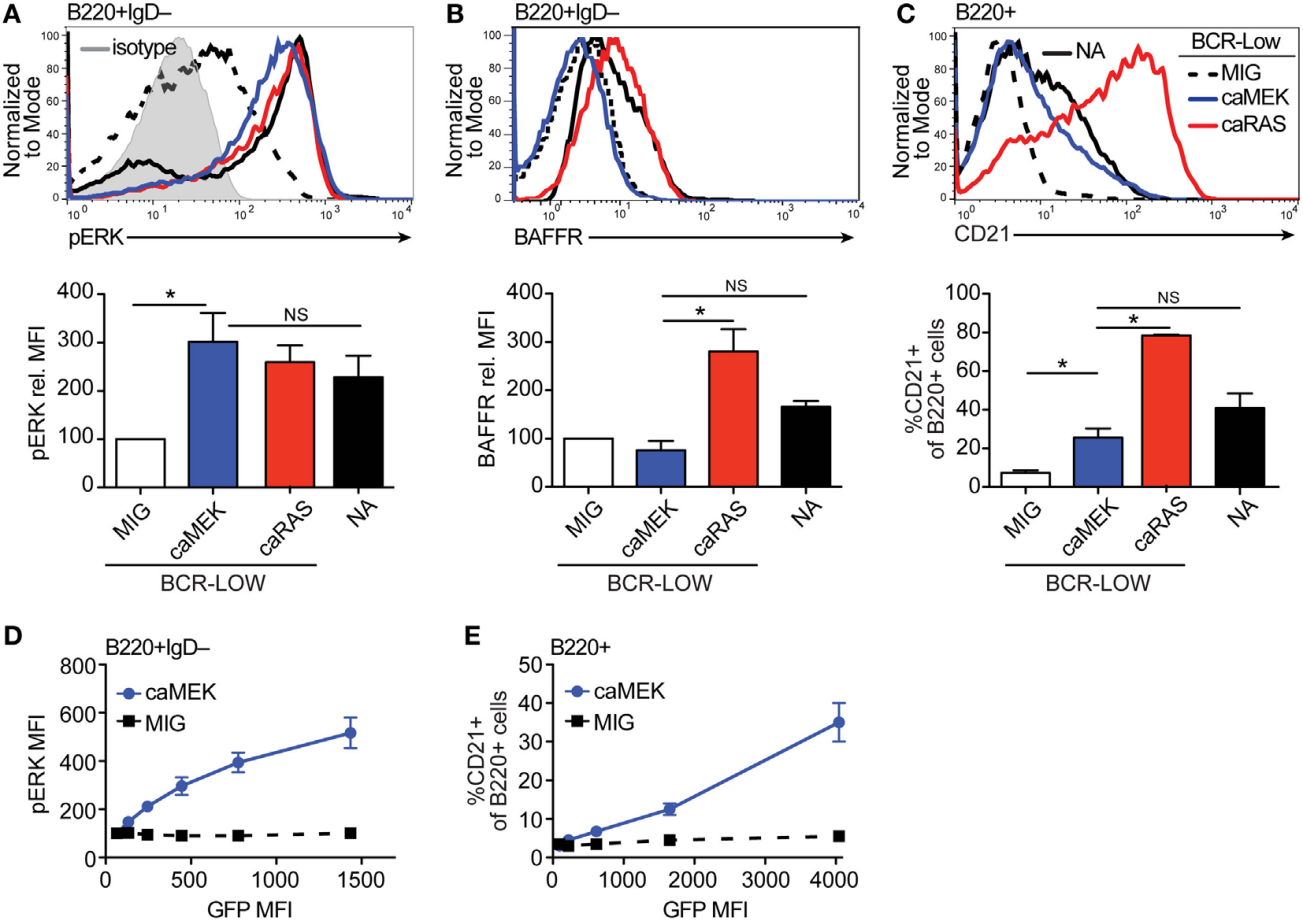
Extracellular signal-regulated kinase (ERK) activation drives differentiation of BCR-low immature B cells *in vitro*. **(A,B)** Representative histograms and bar graph quantification of pERK **(A)** and BAFFR **(B)** in bone marrow immature (B220^+^IgD^–^) B cells cultured for 4 days with IL-7. The cells analyzed were either nonautoreactive (NA) (3-83Igi,H-2^d^) BCR normal cells (NA, black solid line) or BCR-low cells transduced with MIG (black dashed line), caMEK (blue line), or caNRas (red line). The analysis of transduced cells was performed on GFP^+^ cells in all experiments. Staining of pERK was done on cells treated with pervanadate to allow for pERK signal detection. The gray shaded histogram in **(A)** represents isotype control antibody. **(C)** Representative histograms and bar graph quantification of the frequency of CD21^+^ cells in the B220^+^ B cell population after 3 days of culture with BAFF. **(D,E)** BCR-low B cells transduced with either caMEK (blue) or MIG control (black) were gated based on increasing GFP expression, which correlates with caMEK expression in caMEK transduced cells. The pERK MFI **(D)** or the frequency of CD21^+^ cells **(E)** were plotted against the GFP MFI of the individual segments. In all panels, *N* = 3 total, from three independent experiments. *P ≤ 0.05; NS, not significant.

To ensure the function of caMEK, we compared pERK levels in B cells transduced with caMEK or control retrovirus. In order to measure pERK in “naïve” immature B cells, we previously demonstrated the need to amplify this signal with a pervanadate treatment, and we have shown that the pERK signal in pervanadate-treated cells is proportional to the basal level in untreated cells (21). Expression of caMEK in pervanadate-treated BCR-low immature B cells increased pERK levels to those present in BCR-normal NA cells. These levels were also similar to those achieved with the expression of caNRAS (Figure 2A). However, in spite of its ability to increase ERK activity, caMEK failed to increase the expression of BAFFR (Figure 2B). Despite low BAFFR levels, BCR-low immature B cells expressing caMEK differentiated into CD21^+^ transitional B cells similarly to BCR-normal NA B cells, although to a lesser extent relative to BCR-low cells expressing caNRAS (Figure 2C). The ability of caMEK to promote B cell differentiation was dose-dependent because higher levels of caMEK, as indicated by GFP intensity, resulted in higher amounts of pERK and frequency of CD21^+^ cells (Figures 2D,E). Thus, these data confirm our previous findings (20) that immature B cells expressing low levels of BCR display low levels of pERK and BAFFR and do not differentiate into transitional B cells. Furthermore, these data extend these findings to indicate that activation of the ERK pathway restores normal differentiation of immature BCR-low B cells into transitional B cells in culture, but to a lower extent than what was achieved by activation of the RAS pathway.

**Figure 3:**
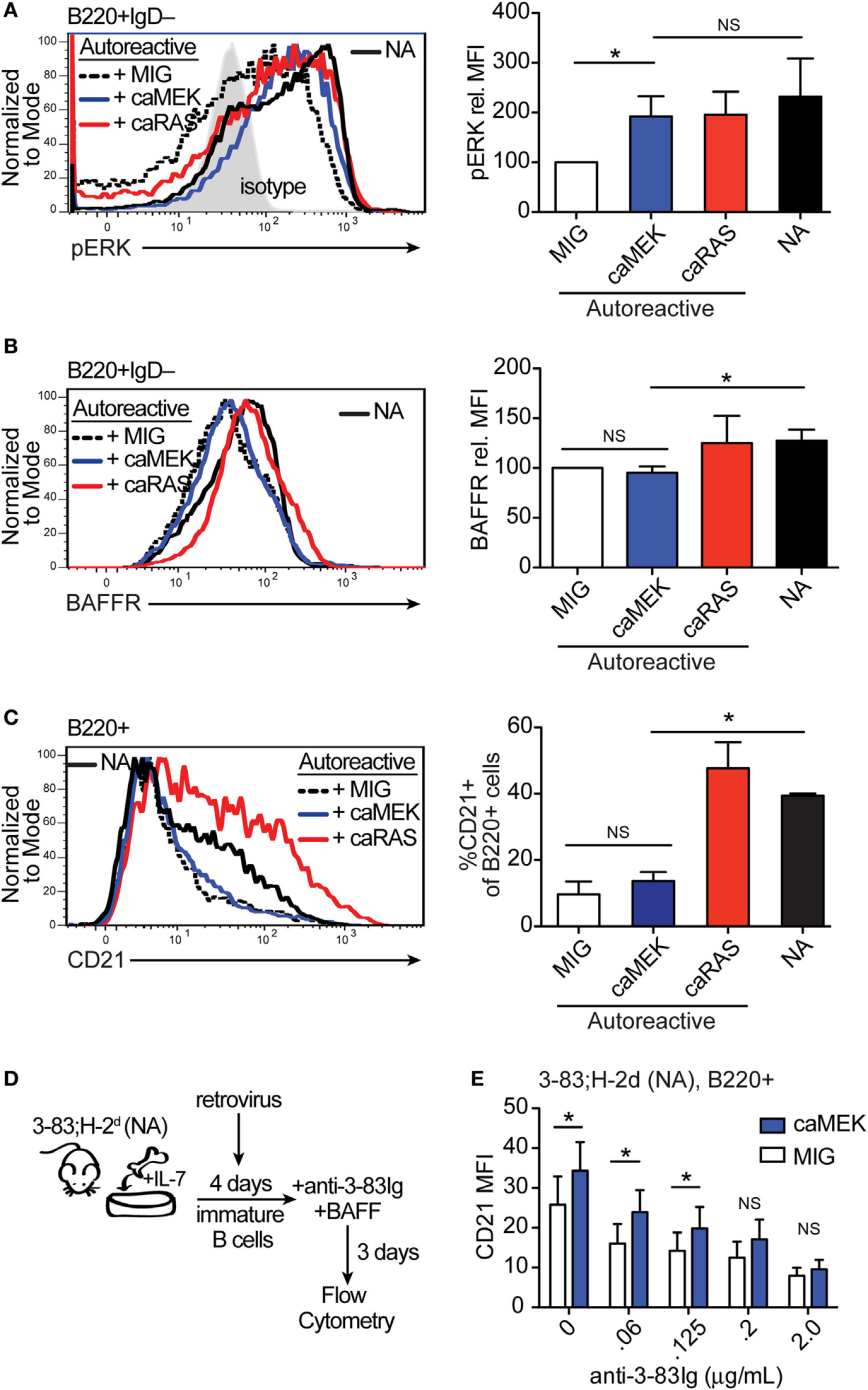
Extracellular signal-regulated kinase (ERK) activation drives the differentiation of low-avidity, but not high-avidity autoreactive B cells *in vitro*. **(A,B)** Representative histograms and bar graph quantification of phospho-ERK (pERK) **(A)** and BAFFR **(B)** in bone marrow immature B cells (B220^+^IgD^–^) cultured for 4 days with IL-7. The B cells analyzed were either non-transduced nonautoreactive (NA) (3-83Igi,H-2^d^) cells (black solid line) or autoreactive (3-83Igi,H-2^b^) cells transduced with MIG control (black dashed line), caMEK (blue line), or caNRas (red line) retroviral vectors. Transduced cells were gated on GFP^+^ for analyses. Staining of pERK (in A) was done on cells treated with pervanadate. The gray shaded histogram in **(A)** represents isotype control antibody. **(C)** Representative histograms and bar graph quantification of the frequency of CD21^+^ cells in the B220^+^ B cell population after 3 days of culture with BAFF. **(D)** Schematic of the system utilized to investigate the effect of caMEK on the *in vitro* differentiation of immature B cells cultured with increasing amounts of BCR stimulation. 3-83Igi,H-2^d^ bone marrow cells were cultured with IL-7 for 4 days and transduced with retrovirus carrying either caMEK or MIG on the second day of culture. Cells were then incubated with BAFF and increasing amounts of an agonistic anti-3-83Ig idiotypic mAb (S23), this latter added each day during a 3 days culture. **(E)** CD21 expression (MFI) on B cells treated as described in **(D)**: caMEK-GFP, blue bars and MIG, white bars. In all panels, *N* = 3 total, from three independent experiments. *P ≤ 0.05; NS, not significant.

We have previously shown that expression of caNRAS promotes the *in vitro* differentiation of high avidity autoreactive immature B cells into transitional B cells and *via* a process requiring the activity of ERK (21). To explore whether direct activation of ERK is sufficient to induce the differentiation of autoreactive B cells, we transduced autoreactive immature B cells (from the 3-83Ig,H-2^b^ mouse model) with caMEK and analyzed their differentiation *in vitro*. Similar to that observed with NA BCR-low B cells, the expression of caMEK in autoreactive B cells led to significantly increased levels of basal pERK, comparable to the levels expressed by NA B cells (Figure 3A). But again, caMEK was unable to increase BAFFR expression (Figure 3B). However, while caMEK corrected the impaired differentiation of BCR-low cells (Figures 2C,E), it did not overcome the developmental block in autoreactive immature B cells (Figure 3C). Indeed, the frequency of CD21^+^ B cells in caMEK-expressing 3-83Ig,H-2^b^ cells was equivalent to that of the control MIG-transduced cells (Figure 3C). These data, therefore, indicate that activation of the MEK-ERK pathway is not sufficient to rescue the differentiation of autoreactive B cells *in vitro*, at least not when the autoreactive cells exhibit high avidity for self-antigen, as is the case for B cells from 3-83Ig,H-2^b^ mice (42).

Autoreactive immature B cells with low avidity for self-antigen experience low levels of self-antigen-induced BCR signaling (43) but also retain a discernable amount of surface IgM and resulting tonic BCR signals (21). In order to test whether constitutive activation of the MEK-ERK pathway could overcome tolerance set by low avidity BCR-induced signals, we used decreasing amounts of the agonistic anti-3-83Ig idiotypic antibody S23 (33) to mimic decreasing availability of self-antigen. Specifically, NA 3-83Igi,H-2^d^ immature B cells were transduced with caMEK or MIG and then incubated with varying concentrations of anti-3-83Ig S23 mAb until analysis of cell differentiation (Figure 3D). Consistent with the previous results (Figure 3C), expression of caMEK did not promote the differentiation of 3-83Ig^+^ B cells incubated with high concentrations of S23 (Figure 3E). However, a measurable and significant increase in cell differentiation was observed in cells expressing caMEK relative to control (MIG) with diminishing doses of S23 (Figure 3E). This suggests that ERK activation may be able to overcome low levels of self-antigen-mediated signals to induce positive selection and differentiation of low-avidity autoreactive immature B cells.

The above findings indicate that activation of the MEK-ERK pathway is able to overcome defective tonic BCR signaling and low level of antigen-induced BCR signaling during the *in vitro* differentiation of immature B cells into transitional B cells and *via* a process independent on the expression of BAFFR.

### Constitutive Activation of the MEK-ERK Pathway Does not Overcome B Cell Tolerance *In Vivo*

**Figure 4:**
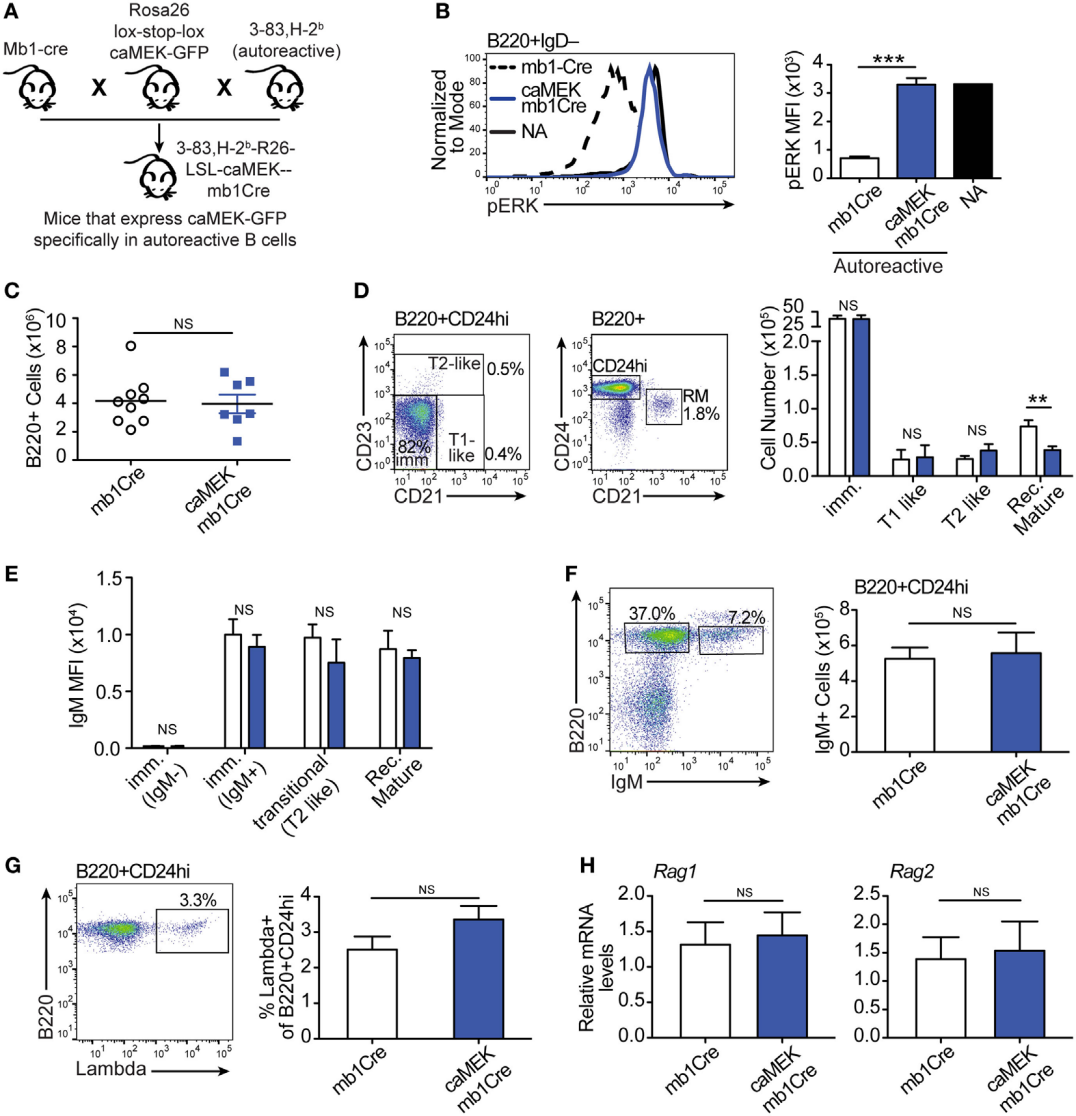
Constitutive extracellular signal-regulated kinase (ERK) activation does not rescue the *in vivo* development of autoreactive bone marrow B cells. **(A)** Schematic of the *in vivo* model for caMEK expression in autoreactive B cells. **(B)** Representative histograms and bar graph quantification of pERK levels in *ex vivo* immature B cells (B220^+^IgD^–^) from the bone marrow of NA (3-83Igi,H-2^d^) mice and autoreactive (3-83Igi,H-2^b^) mb1Cre or R26-LSL-caMEK-GFP-mb1Cre (caMEK-mb1Cre) mice. Cells were treated with pervanadate before pERK staining. **(C)** Absolute numbers of B220^+^ cells in the bone marrow of 3-83Igi,H-2^b^ mb1Cre and R26-LSL-caMEK-GFP-mb1Cre mice. **(D)** Gating strategy and bar graph quantification of cell numbers in bone marrow B cell populations from 3-83Igi,H-2^b^ mb1Cre and R26-LSL-caMEK-GFP-mb1Cre mice. B cell subsets were discriminated as: immature B cells (B220^+^CD24^hi^CD21^–^CD23^–^), T1-like B cells (B220^+^CD24^hi^CD21^+^CD23^–^), T2-like B cells (B220^+^CD24^hi^ CD21^±^CD23^+^), and recirculating mature B cells (B220^+^CD24^lo^CD21^hi^). **(E)** Mean fluorescence intensity of IgM surface expression on B cells belonging to the B cell subsets gated as in **(D)**. **(F)** Gating strategy and quantification of the number of IgM^+^ (edited) and IgM^–^ (editing) cells within the bone marrow immature (B220^+^CD24^hi^) B cell population from 3-83Igi,H-2^b^ mb1Cre and R26-LSL-caMEK-GFP-mb1Cre mice. **(G)** Gating strategy and quantification of the percentage of λ+ cells within immature (B220^+^CD24^hi^) bone marrow B cells from 3-83Igi,H-2^b^ mb1Cre and R26-LSL-caMEK-GFP-mb1Cre mice. For panels **(A–F)**, *N* = 9 total 3-83Igi,H-2^b^-mb1Cre mice and *N* = 7 total 3-83Igi,H-2^b^-R26-LSL-caMEK-GFP-mb1Cre mice, analyzed in four independent experiments. **(H)** Relative *Rag1* and *Rag2* mRNA levels in B220^+^ or B220^+^GFP^+^ cells sorted from the bone marrow of 3-83Igi,H-2^b^-mb1Cre or 3-83Igi,H-2^b^-R26-LSL-caMEK-GFP-mb1Cre mice, respectively. Data were normalized to 18 s mRNA levels and are expressed as fold change over the average mRNA levels in 3-83Igi,H-2^b^-mb1Cre cells. *N* = 3 mice from one experiment. In all panels, B cells from 3-83Igi,H-2^b^ R26-LSL-caMEK-GFP-mb1Cre mice were additionally gated on GFP^+^ to analyze only the cells expressing caMEK. **P ≤ 0.01, ***P ≤ 0.001, NS, not significant.

The bone marrow culture system only partially reflects the processes of central B cell selection and B cell differentiation and cannot be used to investigate the generation of mature B cells or the implementation of peripheral tolerance. To test whether activation of the ERK pathway can overcome, at least partially, central and peripheral B cell tolerance *in vivo*, we crossed 3-83Igi,H-2^b^ mice to mb1-Cre mice (30) and Rosa26-lox-stop-lox-caMEK-GFP mice (25) to generate 3-83Igi,H-2^b^ LSL-caMEK-mb1Cre animals in which caMEK is only expressed in 3-83 autoreactive B cells (Figure 4A).

Expression of caMEK, which was marked by the presence of GFP, was exclusively observed in B220^+^ B cells in the bone marrow and spleen of LSL-caMEK-mb1Cre mice (Figure S2 in Supplementary Material). To confirm the functionality of the Rosa26 caMEK allele in the B cell lineage, we measured pERK levels in bone marrow immature B cells in which the pERK signal was amplified with pervanadate treatment. Bone marrow immature B cells are normally identified as B220^+^IgM^+^IgD^–^, but we analyzed pERK in B220^+^IgD^–^ cells because IgM is largely internalized in 3-83Ig^+^ autoreactive B cells, and about 90% of B220^+^IgD^–^ cells are nevertheless immature B cells in (3-83) Ig knock-in mice due to the absence of pre-B cells (26). The expression of caMEK in autoreactive immature B cells increased their pERK levels to those observed in NA B cells (Figure 4B), and this was not a consequence of differences in surface IgM (Figure 4E) in the presence of pervanadate treatment, as could be argued based on previous studies (44). Thus, these data indicate that caMEK expression in developing B cells leads to meaningful activation of the ERK pathway.

Central B cell tolerance was investigated *in vivo* by first analyzing the phenotype of bone marrow B cells. As shown in Figures 4C,D, activation of the ERK pathway did not alter the total number of B220^+^ cells, nor the numbers of immature (CD21^–^CD23^–^), T1-like (CD21^+^CD23^–^), and T2-like (CD21^+^CD23^+^) B cell populations in the bone marrow of 3-83Igi,H-2^b^ mice. Surface levels of IgM were also equivalent (Figure 4E). However, we did find a slight but significant reduction in numbers of recirculating mature (CD24^low^CD21^+^) B cells (Figure 4D).

It was previously indicated that the ERK pathway plays a role in receptor editing (45), although our previous *in vitro* studies suggest it does not (21). In accordance with our previous findings, we did not see changes in parameters associated with receptor editing such as the number of surface IgM^–^ (editing) and IgM^+^ (edited) cells, frequency of λ^+^ cells, and expression of *Rag*1/2 genes, within the bone marrow immature B cell population (Figures 4F–H).

**Figure 5:**
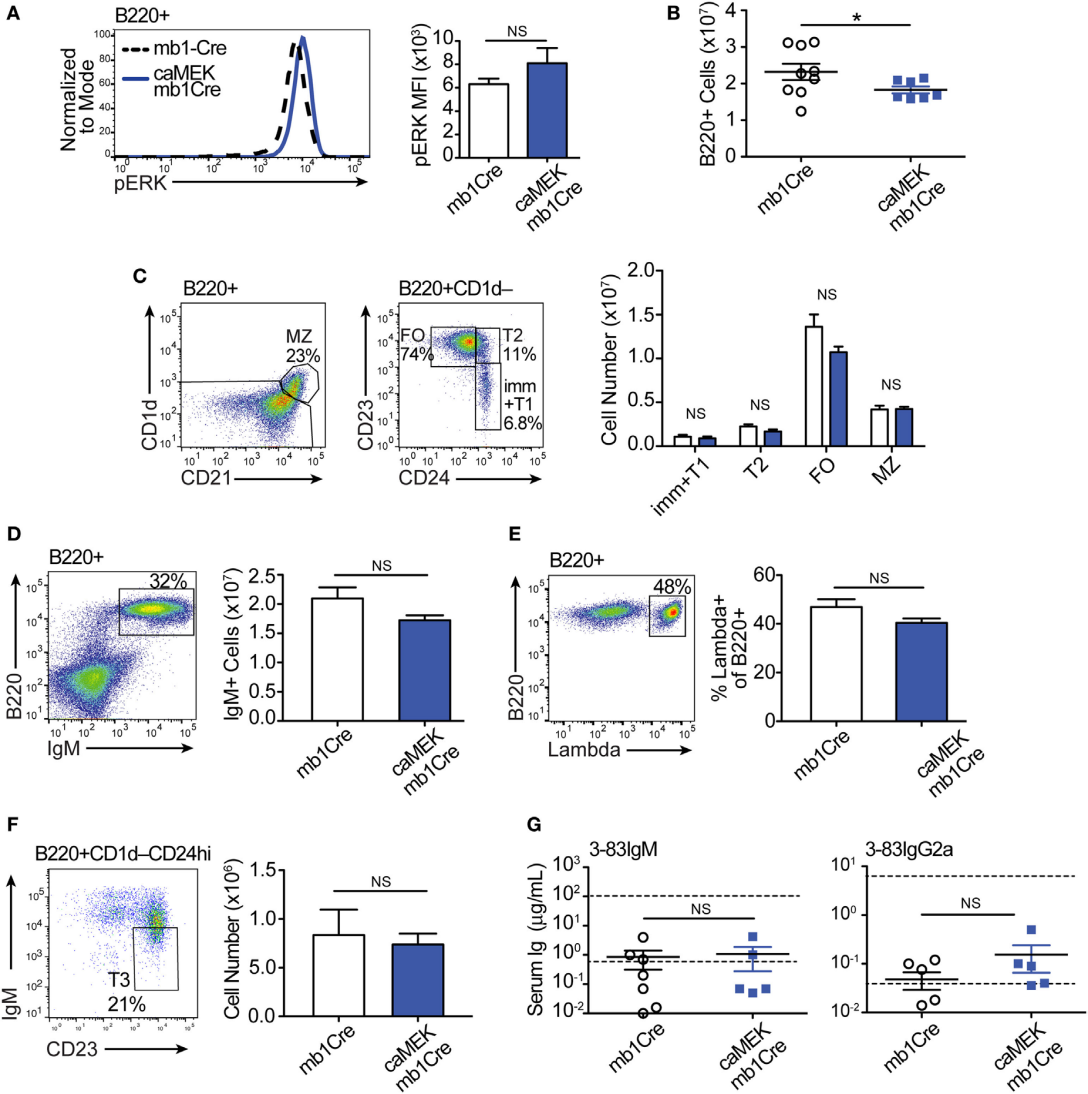
Constitutive extracellular signal-regulated kinase (ERK) activation does not break the central or peripheral tolerance of autoreactive 3-83Ig^+^ B cells *in vivo*. **(A)** Representative histograms and bar graph quantification of phospho-ERK (pERK) levels in *ex vivo* B220^+^ B cells from the spleen of autoreactive 3-83Igi,H-2^b^ mb1Cre and R26-LSL-caMEK-GFP-mb1Cre mice. Cells were treated with pervanadate before pERK staining. **(B)** Absolute number of B220^+^ cells in the spleens of 3-83Igi,H-2^b^ mb1Cre and R26-LSL-caMEK-GFP-mb1Cre mice. **(C)** Gating strategy and bar graph quantification of splenic B cell populations from 3-83Igi,H-2^b^ mb1Cre and R26-LSL-caMEK-GFP-mb1Cre mice. B cell subsets were discriminated as: immature/transitional 1 B cells (B220^+^CD1d^–^CD24^hi^CD23^–^), transitional 2 B cells (B220^+^CD1d^–^CD24^hi^CD23^+^), follicular B cells (B220^+^CD1d^–^CD24^lo^CD23^+^), and marginal zone B cells (B220^+^CD1d^+^CD21^hi^). **(D)** Gating strategy and quantification of IgM^+^ B cell numbers from the spleens of 3-83Igi,H-2^b^ mb1Cre and R26-LSL-caMEK-GFP-mb1Cre mice. **(E)** Gating strategy and quantification of the percentage of λ^+^ cells within B220^+^ B cells from the spleen of 3-83Igi-H-2^b^ mb1Cre and R26-LSL-caMEK-GFP-mb1Cre mice. For panels **(A–E)**, *N* = 9 total 3-83Igi,H-2^b^-mb1Cre mice, and *N* = 7 total 3-83Igi,H-2^b^-R26-LSL-caMEK-GFP-mb1Cre mice, analyzed in four independent experiments. **(F)** Gating strategy and quantification of T3 B cell numbers (CD23^+^IgM^lo^) within the transitional B cell population (B220^+^CD1d^–^CD24^hi^) from the spleen of 3-83Igi,H-2^b^ mb1Cre and R26-LSL-caMEK-GFP-mb1Cre mice. In panels **(A–F)**, B cells from 3-83Igi,H-2^b^ R26-LSL-caMEK-GFP-mb1Cre mice were additionally gated on GFP^+^ to analyze only the cells expressing caMEK. **(G)** Concentration (μg/mL) of 3-83IgM and 3-83IgG2a in the sera of 3-83Igi,H-2^b^-mb1Cre controls (*N* = 7) and 3-83Igi,H-2^b^-R26-LSL-caMEK-GFP-mb1Cre mice (*N* = 5). The bottom dotted lines represent background detection levels from a wild-type control and the top dotted lines represent Ig levels present in 3-83,H-2^d^ nonautoreactive (NA) positive control mice. *P ≤ 0.05; NS, not significant.

We next evaluated the extent to which activation of the ERK pathway affects B cell tolerance by analyzing the phenotype of peripheral B cells. In the spleen of 3-83Igi,H-2^b^ caMEK-mb1Cre mice, we noticed a slight, although not significant, increase in pERK expression (Figure 5A). We also observed a minor, but significant reduction in total B220^+^ cell numbers (Figure 5B), but there were no other significant differences in individual splenic B cell subsets compared to mb1Cre only littermate controls (Figure 5C). There were also no differences in IgM^+^ cell numbers or in the percent of λ^+^ B cells between caMEK and control mice (Figures 5D,E), confirming our conclusions that central B cell tolerance was equivalent in its extent or mechanism. Because ERK signaling has been thought to maintain anergy in a subset of autoreactive B cells (43), we analyzed the T3 B cell subset to investigate whether expression of caMEK leads to increased numbers of anergic B cells (6). However, we found no difference in the T3 population of caMEK mice compared to controls (Figure 5F). In the past, we have shown that about 20% of splenic B cells in 3-83Igi,H-2^b^ mice retains expression of the 3-83 autoreactive BCR, although this is generally intracellular and the antibody is minimally secreted (29). Thus, we questioned next whether caMEK would relax peripheral tolerance and increase secretion of the 3-83 autoantibody. Again, we observed no significant differences in the concentrations of 3-83IgM and 3-83IgG2a secreted antibodies in serum of caMEK and control mice, antibodies that were detected only at background (wild-type) levels (Figure 5G).In summary, these data indicate that caMEK expression by high avidity autoreactive B cells does not lead to a break in either central or peripheral tolerance *in vivo*.

To establish whether the inability of caMEK to correct defects in B cell differentiation *in vivo* was restricted to autoreactive B cells, we also investigated the effect of caMEK on the *in vivo* development of NA BCR-low B cells. For these studies, we generated bone marrow chimeras of Igh^a^ BCR-low cells transduced with either caMEK-GFP or GFP only, mixed with non-transduced Igh^b^ wild-type competitor cells (Figure S3A in Supplementary Material) using a method previously described (20). We found that although ERK activation was increased, BCR-low B cells expressing caMEK were unable to differentiate *in vivo* (Figures S3B–E in Supplementary Material), similar to that observed for autoreactive B cells.

Taken together, these data indicate that when assessed *in vivo*, direct activation of the ERK pathway is unable to compensate for defective tonic BCR signaling or self-antigen-induced BCR signals during B cell development and tolerance.

### Constitutive Activation of the ERK Pathway in B Cells Leads to Higher Levels of Serum Antibodies but Not to Higher Numbers of Activated B Cells

Published studies indicate that ERK is necessary for the expression of BLIMP-1 and the generation of plasma cells in response to foreign antigen (46). However, continuous BCR signaling through the ERK pathway, as present in self-reactive B cells, leads to inhibition of BLIMP-1 and plasma cell generation *via* a mechanism that has been suggested to be necessary for the maintenance of B cell anergy (43, 47). We were therefore interested in whether constitutive activation of the MEK-ERK pathway has either a positive or negative impact on B cell activation and antibody production.

**Figure 6:**
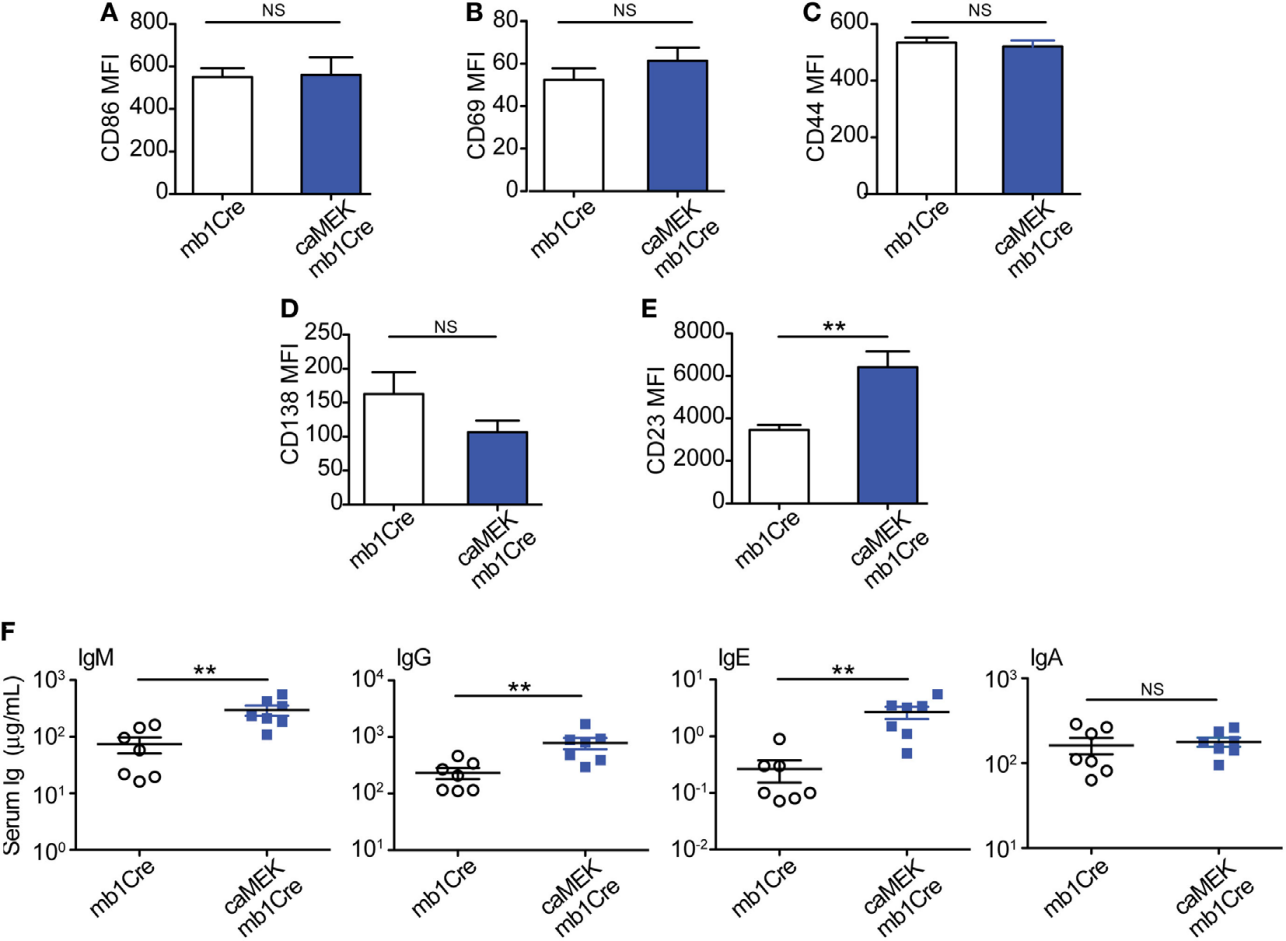
B cell-specific expression of caMEK does not induce B cell activation, but increases serum Ig levels. **(A–E)** MFI levels of **(A)** CD86, **(B)** CD69, **(C)** CD44, **(D)** CD138, and **(E)** CD23 on splenic B220^+^ B cells from 3-83Igi, H-2^b^ mb1Cre and B220^+^GFP^+^ B cells R26-LSL-caMEK-GFP-mb1Cre mice. *N* = 9 total 3-83Igi,H-2^b^-mb1Cre mice and *N* = 7 total 3-83Igi,H-2^b^-R26-LSL-caMEK-GFP-mb1Cre mice, analyzed in four independent experiments. **(F)** Concentrations (μg/mL) of total IgM, IgG, IgE, and IgA in the sera of 3-83Igi,H-2^b^-mb1Cre littermate controls (*N* = 7, open circles) and 3-83Igi,H-2^b^-R26-LSL-caMEK-GFP-mb1Cre (*N* = 7, filled squares) mice. **P ≤ 0.01; NS, not significant.

To address this, we evaluated naïve 3-83Igi,H-2^b^ caMEK and control mice for the expression of activation markers on B cells and the levels of total serum Igs, taking into consideration that the peripheral B cell population of 3-83Igi,H-2^b^ mice is mainly composed of edited polyclonal NA B cells (28, 29). Our analyses did not detect any significant differences in the expression of CD86 and CD69 activation markers and in that of CD44 and CD138 plasmablast markers on caMEK splenic B cells, relative to control (Figures 6A–D). We did, however, find a significant increase of CD23 surface levels in caMEK B cells (Figure 6E). CD23 is known to be a receptor for IgE, and previous studies have reported that IgE is able to regulate the expression of CD23 *in vivo* (48). Based on this and the known contribution of ERK to plasma cell development (46), we hypothesized that constitutive activation of the MEK-ERK pathway could alter antibody production, and we tested this possibility by measuring total Ig isotypes in the sera of caMEK and control mice. These analyses detected significantly higher levels of total IgM, IgG, and IgE, but not IgA, in caMEK mice compared to littermate controls (Figure 6F).

Thus, by showing that B cell-specific constitutive activation of the MEK-ERK pathway increases basal antibody production, these data support a B cell-intrinsic role of ERK in plasma cell generation and antibody secretion.

## Discussion

The present study extends our understanding of how central B cell tolerance is intrinsically regulated and how autoreactive B cells are selected out of the bone marrow and into the circulation, this latter a phenomenon that occurs more frequently in many autoimmune patients. We have previously established that activation of the RAS cascade can break central B cell tolerance in mice, leading to inhibition of receptor editing and development of autoreactive B cells, a process requiring ERK. In this study, we tested whether intrinsic activation of the ERK pathway in immature B cells is not only necessary, but also sufficient to mediate BCR-derived signaling events that promote cell differentiation and positive selection into the mature population. Our data demonstrate that constitutive activation of the MEK-ERK pathway can intrinsically overcome suboptimal levels of tonic BCR signaling and low amounts of antigen-induced BCR signaling to promote the differentiation of immature B cells into transitional B cells, but only *in vitro*. *In vivo*, however, activation of the MEK-ERK pathway is unable to break either central or peripheral B cell tolerance, despite its ability to generally enhance serum antibody levels.

The RAS-ERK pathway has been positively linked to a variety of processes during B cell development. Starting at the very early B cell subsets, it has been shown that ERK activity is essential for the pro-B cell to pre-B cell transition (49, 50). Furthermore, the ERK kinase signals downstream of the pre-BCR to upregulate genes associated with cell proliferation and survival (50). In contrast to these positive effects exercised during early B cell development, ERK can have quite a negative effect during the transitional B cells stage. In fact, it has been shown that B cells can activate the ERK pathway both dependently and independently of Ca^2+^ signaling, and that Ca^2+^-dependent activation of the ERK pathway is responsible for inducing apoptosis (i.e., clonal deletion) of autoreactive B cells at the transitional stage (51).

In this study we show that, differing from what was previously achieved with constitutive activation of RAS (20, 21), direct ERK activation is not sufficient to induce positive selection and bone marrow export of either NA BCR-low or autoreactive B cells. We additionally show that basal activation of the ERK pathway, such as achieved in the 3-83Igi,H-2^b^,caMEK,mb1-cre mice, does not induce general apoptosis of transitional B cells. This is evident by the overall normal B cell maturation observed in 3-83Igi,H-2^b^,caMEK,mb1-cre mice. Thus, our data disagree with a general pro-apoptotic role of ERK in transitional B cells (51). We did notice, however, a slight reduction in recirculating mature B cells in the bone marrow and of total B cells in the spleen of the caMEK mice (Figures 4D and 5B). Therefore, it is possible that these changes are caused by mechanisms of negative selection resulting from a small subset of caMEK-positive B cells acquiring greater than basal levels of ERK activation and subsequently undergoing clonal deletion. Clearly, other possibilities exist such as ERK mediated alteration of cell trafficking.

This study, as well as our previous study (21), convincingly shows that the ERK pathway does not regulate *Rag1/*2 expression and, thus, receptor editing, in autoreactive immature B cells. These findings contrast with published studies from other groups suggesting that ERK is able to inhibit receptor editing (45, 52). Although the basis for this discrepancy is not clear, we note that in our experiments caMEK increased pERK in (pervanadate-treated) autoreactive cells to levels similarly expressed by NA immature B cells and, thus, reflecting normal physiology. The other studies either used a B cell lymphoma (52) which, as a transformed cell, possibly displays aberrant signaling properties, or employed ERK activated by a constitutively active form of RAF (45). Because in this latter study pERK levels were not reported, we cannot exclude the possibility they were higher than those promoted by caMEK in our studies, leading to distinct B cell fates.

Throughout our studies, we observed some discrepancies between the results we obtained from the *in vitro* B cell cultures and the *in vivo* mouse models. Namely, we saw that activation of the MEK-ERK pathway was able to promote differentiation of BCR-low cells in bone marrow cell cultures but not in mixed retrogenic bone marrow chimeras. There could be several reasons for this discrepancy. One of these is based on the knowledge that autoreactive B cells are better able to thrive in *in vitro* B cell culture systems than in *in vivo* mouse models (53), and this could also be true for BCR-low NA immature B cells. We also noticed that, although necessary, ERK activation is not sufficient to induce BAFFR expression on immature B cells. It may be argued that the generation of transitional B cells is more dependent on BAFFR expression and signaling *in vivo* than *in vitro*. BAFF has been shown to mediate B cell survival *via* ERK but also *via* PI3K-AKT (54, 55). Thus, expression of caMEK may relieve the requirement for BAFF-induced ERK activation *in vitro*, but not *in vivo* where activation of the PI3K-AKT pathway may be needed because of heightened cell competition. Overall, our data suggest that ERK activation can overcome the requirement for tonic BCR and BAFFR signaling in the differentiation of immature B cells into transitional B cells *in vitro*, but not *in vivo*.

Because phospho-ERK levels are elevated in anergic B cells, it has been proposed that ERK activation may be responsible for the induction or maintenance of anergy (6, 43). However, whether ERK activation alone is able to induce a state of B cell anergy has never been directly tested. Our data show that there was no difference in the size of the T3 B cell population in caMEK and control mice, suggesting that B cell-intrinsic ERK activation alone may not be sufficient to induce an anergic phenotype. It must however be noted that in these previous studies (6, 43, 46, 47, 49) pERK levels were measured by Western Blot and were typically not quantified. Therefore, we cannot exclude that quantitative differences in ERK activation between these and our studies are responsible for the differences observed in B cell anergy. Additional studies have suggested that constitutive activation of the ERK pathway is responsible for inhibiting antibody responses to TLR agonists in autoreactive B cells (47, 49). However, seemingly conflicting findings based on the use of conditional ERK deficient mice have shown that ERK activity is indispensable for BLIMP expression, generation of plasma cells, and T cell-dependent antibody responses (46). Although these data initially seem to be contradictory, it is possible that ERK activation in B cells produces different outcomes at distinct stages of differentiation (i.e., transitional versus follicular B cells), during T cell-independent vs. T cell-dependent antibody responses, or whether the B cells are autoreactive or not. Indeed, our data seem to support this idea by demonstrating that continuous activation of the ERK pathway in B cells of naïve mice enhances basal antibody production but does not promote autoantibody secretion.

It was surprising to find that constitutive activation of MEK-ERK did not lead to general B cell activation, or to changes in numbers of marginal zone B cells whose development is regulated by BCR signaling (56). Despite a lack of general B cell activation, CD23 expression on B cells and levels of IgE in serum of caMEK mice were both elevated. It has previously been reported that IgE and CD23 regulate each other. Specifically, CD23 transgenic mice produce lower IgE (57), while B cells of IgE transgenic mice show CD23 upregulation (58). Given we observed increase of both CD23 and IgE as well as higher levels of other Ig isotypes (IgM, IgG), we conclude that B cell-intrinsic expression of caMEK leads first to higher IgE production which in turn positively regulates CD23 expression on B cells. As mentioned above, mice with conditional deletion of ERK in B cells indicate ERK’s requirement for plasma cell generation (46). Our data showing enhanced serum Ig levels in caMEK mice support this conclusion.

Overall, our data indicate there are significant differences in the individual abilities of the RAS and ERK pathways in mediating B cell selection and maturation both *in vitro* and *in vivo*. ERK is often thought of as the main signaling mediator downstream of RAS. However, our findings clearly show that while basal activation of the MEK-ERK pathway in immature B cells is necessary for the upregulation of BAFFR and the generation of transitional B cells, at least *in vitro*, activation of this pathway *in vivo* is not sufficient for overcoming defects in tonic BCR signaling and for breaking B cell tolerance, both in the bone marrow and in the periphery. Thus, these results argue that there are parallel pathways downstream of RAS and distinct from MEK-ERK signaling that converge to induce BAFFR expression, inhibition of receptor editing, and positive selection of developing B cells and that may additionally regulate autoreactive B cell generation in autoimmunity. We have previously shown that inhibition of PI3K also diminishes, to some extent, the cell differentiation induced by RAS activation in immature B cells. In addition, PI3K is required for the RAS-mediated inhibition of receptor editing (21). This suggests that PI3K may be the pathway through which RAS mediates crucial signals in immature B cell selection and maturation and we are currently exploring this possibility.

## Ethics Statement

All animal procedures were approved by the UCD Institutional Animal Care and Use Committee.

## Author Contributions

SG, RP, and RT contributed conception and design of the study; SG performed all experiments with the technical assistance of JP. SG analyzed data and drew conclusions together with RP. SG wrote the first draft of the manuscript. SG, RP, and RT contributed to manuscript revision, read and approved the submitted version.

## Conflict of Interest Statement

The authors declare that the research was conducted in the absence of any commercial or financial relationships that could be construed as a potential conflict of interest.
