# Corrigendum: Activation of the MEK-ERK Pathway Is Necessary but Not Sufficient for Breaking Central B Cell Tolerance

**DOI:** 10.3389/fimmu.2018.02218

**Published:** 2018-09-27

**Authors:** Sarah A. Greaves, Jacob N. Peterson, Raul M. Torres, Roberta Pelanda

**Affiliations:** ^1^Department of Immunology and Microbiology, University of Colorado School of Medicine, Aurora, CO, United States; ^2^Department of Biomedical Research, National Jewish Health Denver, CO, United States

**Keywords:** B cells, B cell tolerance, BCR signaling, MAP kinase, ERK, B cell development, autoreactive B cells, mouse models

In the original article, a mistake was found in affiliation 1. The state should be CO (Colorado) instead of IL (Illinois). The authors apologize for this error and state that this does not change the scientific conclusions of the article in any way.

The original article has been updated.

## Conflict of interest statement

The authors declare that the research was conducted in the absence of any commercial or financial relationships that could be construed as a potential conflict of interest.

