# Supplementary material for: Activation of the MEK-ERK Pathway Is Necessary but Not Sufficient for Breaking Central B Cell Tolerance
Source: Front Immunol. 2018 Apr 9;9:707. doi: 10.3389/fimmu.2018.00707 (PMC5900439; doi:10.3389/fimmu.2018.00707)
Supplement: Supplementary file 1 [file data_sheet_1.PDF]

Supplementary Material

Activation of the MEK-ERK pathway is necessary but not sufficient for breaking central B cell tolerance

Sarah A. Greaves[1], Jacob N. Peterson[1], Raul M. Torres[1,2], and Roberta Pelanda[1,2]

[1]Department of Immunology and Microbiology, University of Colorado Denver School of Medicine, Aurora, CO 80045, USA

[2]Department of Biomedical Research, National Jewish Health, Denver, CO 80206, USA

Corresponding Author: Roberta Pelanda: Roberta.Pelanda@ucdenver.edu

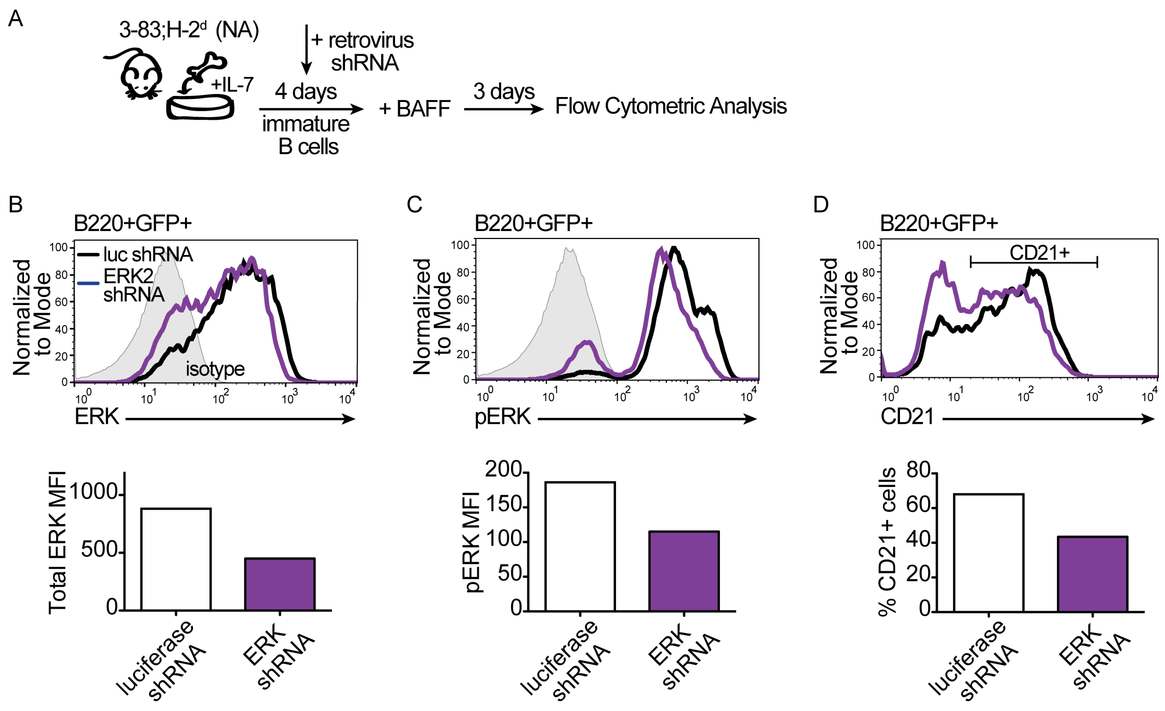

Supplemental Figure 1. ERK contributes to the *in vitro* differentiation of immature B cells into the transitional stage. (A) Schematic of the system to study immature B cell differentiation *in vitro*. Bone marrow cells isolated from 3-83Igi mice were cultured with IL-7 for a total of 4 days. On the second day of culture, cells were transduced with retrovirus containing either ERK specific shRNA or control luciferase shRNA, both also expressing GFP. Cells were then incubated without IL-7 and with BAFF for additional 3 days to promote cell differentiation. (B,C) Representative histograms and bar graph quantification of total ERK (B) and pERK (C) measured in GFP^+ nonautoreactive (3-83Igi,H-2^d) immature B cells ($B220^+IgD^-$) 2 days after transduction with luciferase shRNA (black) or ERK shRNA (purple). Cells stained for pERK were first treated with the phosphatase inhibitor pervanadate to allow for signal detection. The gray shaded histograms are cells stained with isotype control antibody. (D) Representative histograms and bar graph quantification of the frequency of $CD21^+$ cells in the $B220^+$ B cell population of transduced (GFP^+) cells after 3 days of culture with BAFF. (B-D) N=2 total from 2 independent experiments.

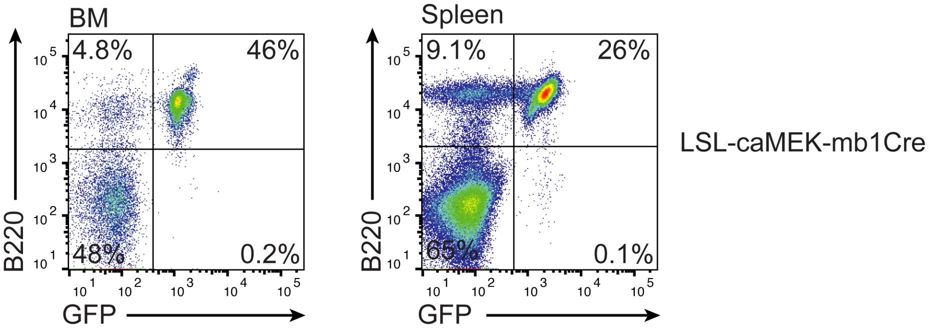

Supplemental Figure 2. Monitoring caMEK expression by the expression of GFP in B cells of mice. Representative dot plots showing expression of GFP vs. B220 in bone marrow and spleen lymphocytes of 3-83Igi,H-2^b-R26-LSL-cMEK-GFP-mb1Cre mice. Numbers indicate frequency of cells in each quadrant.

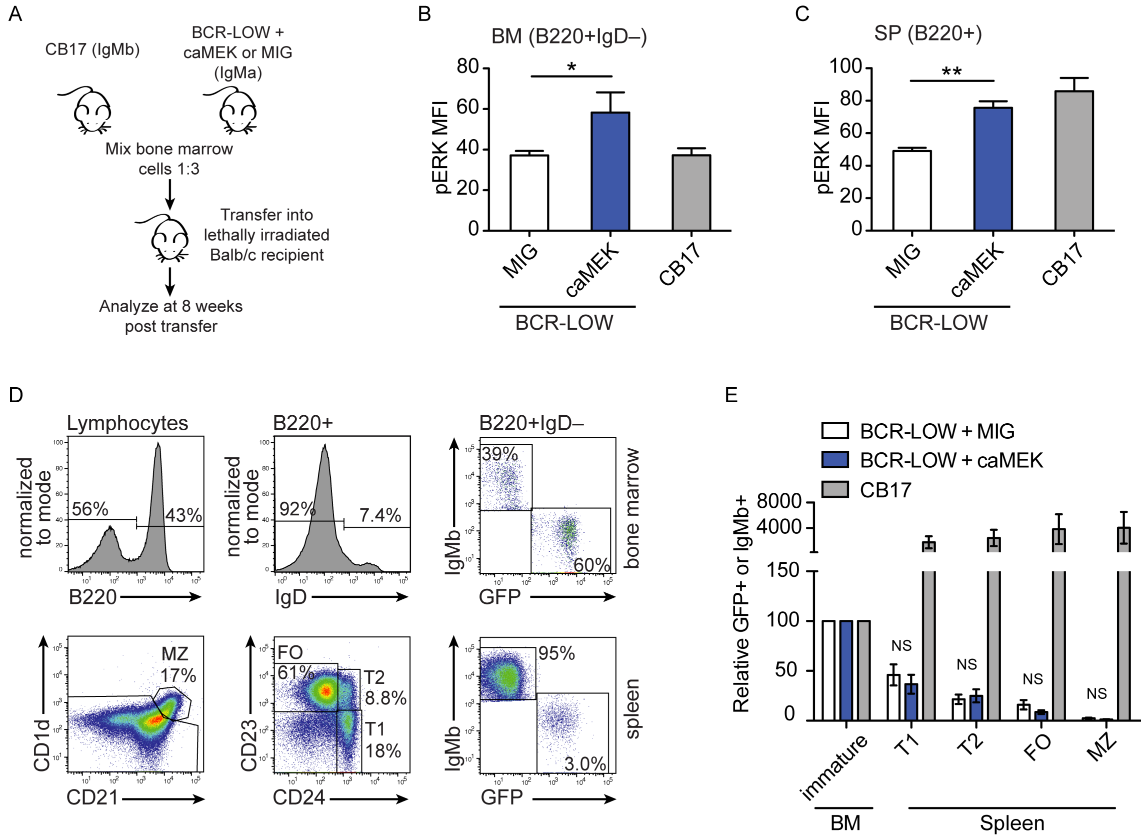

Supplemental Figure 3. Competitive development of BCR-low B cells expressing caMEK. (A) Schematic for the generation of mixed bone marrow chimeras generated with a mixture of non-transduced CB17 bone marrow cells with caMEK or MIG transduced BCR-low bone marrow cells. (B,C) Bar graph quantification of pERK levels (MFI) in (B) immature bone marrow cells (B220^{+}IgD^{-}) and (C) splenic B220^{+} B cells from mixed bone marrow chimeras generated as described in (A). BCR-low transduced cells were also gated as GFP^{+} to include in the analysis only transduced cells. (D) Flow cytometric analysis and gating strategy of bone marrow and spleen cells from the mixed bone marrow chimeras. Bone marrow immature B cells (top row plots) were gated as B220^{+} and IgD^{-} (representative histograms) and then analyzed for IgM^{b} and GFP (pseudocolor plot in top row) to discriminate IgM^{b} CB17 wild-type cells and GFP^{+} transduced BCR-low cells. Spleen B220^{+} B cells (bottom row plots) were analyzed for CD1d vs. CD21 (bottom left plot) and CD23 vs. CD24 (bottom middle plot) to discriminate the following B cell subsets: marginal zone B cells (B220^{+}CD1d^{+}CD21^{hi}), transitional 1 B cells (B220^{+}CD1d^{-}CD24^{hi}CD23^{-}), transitional 2 B cells (B220^{+}CD1d^{-} CD24^{hi}CD23^{+}), and follicular B cells (B220^{+}CD1d^{-}CD24^{lo}CD23^{+}). Splenic B cell subsets were then analyzed for IgM^{b} and GFP (bottom right plot) to discriminate IgM^{b} CB17 wild-type cells and GFP^{+} transduced BCR-low cells. (E) B cell populations (either IgM^{b} or GFP^{+}) in the spleen of chimeras normalized to the respective immature population in the bone marrow (set at 100 in the graph). N=6 for both BCR-low+MIG and BCR-low+caMEK-GFP cells and N=12 for CB17 cells (total 12 mice), from one experiment. $*P \leq 0.05$, $**P \leq 0.01$.
